# Guanidinium phenyl­arsonate–guanidine–water (1/1/2)

**DOI:** 10.1107/S1600536810025043

**Published:** 2010-07-03

**Authors:** Graham Smith, Urs D. Wermuth

**Affiliations:** a Faculty of Science and Technology, Queensland University of Technology, GPO Box 2434, Brisbane, Queensland 4001, Australia

## Abstract

In the structure of the title compound, CH_6_N_3_^+^·C_6_H_6_AsO_3_^−^·CH_5_N_3_·2H_2_O, the phenyl­arsonate anion participates in two *R*_2_^2^(8) cyclic hydrogen-bonding inter­actions, one with a guanidinium cation, the other with a guanidine mol­ecule. The anions are also bridged by the water mol­ecules, one of which completes a cyclic *R*_5_^3^(9) hydrogen-bonding association with the guanidinum cation, conjoint with one of the three *R*_2_^2^(8) associations about that ion, as well as forming an *R*_2_^1^(6) cyclic association with the guanidine mol­ecule. The result is a three-dimensional framework structure.

## Related literature

For chemical data on phenyl­arsonic acid, see: O’Neil (2001 ▶). For related guanidinium structures, see: Smith *et al.* (2001 ▶); Smith & Wermuth (2010 ▶); Sun *et al.* (2002 ▶); Swift & Ward (1998 ▶); Swift *et al.* (1998 ▶); Mak & Xue (2000 ▶). For graph-set analysis, see: Etter *et al.* (1990 ▶).
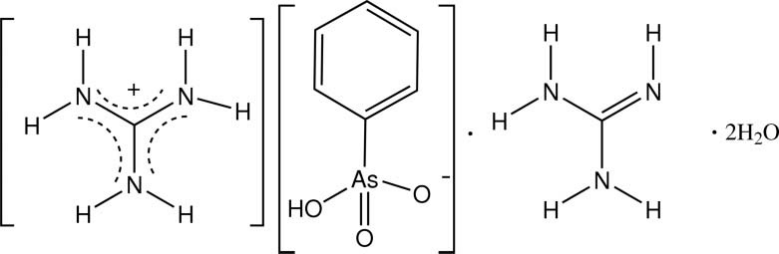


## Experimental

### 

#### Crystal data

CH_6_N_3_^+^·C_6_H_6_AsO_3_^−^·CH_5_N_3_·2H_2_O
*M* *_r_* = 356.23
Monoclinic, 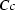

*a* = 18.6545 (14) Å
*b* = 7.6394 (3) Å
*c* = 12.6319 (10) Å
β = 121.856 (10)°
*V* = 1529.0 (2) Å^3^
*Z* = 4
Mo *K*α radiation
μ = 2.25 mm^−1^
*T* = 200 K
0.27 × 0.25 × 0.20 mm

#### Data collection

Oxford Diffraction Gemini-S CCD-detector diffractometer
Absorption correction: multi-scan (*SADABS*; Sheldrick, 1996 ▶) *T*_min_ = 0.935, *T*_max_ = 0.985
4919 measured reflections
2095 independent reflections
1940 reflections with *I* > 2σ(*I*)
*R*_int_ = 0.024

#### Refinement

*R*[*F* ^2^ > 2σ(*F* ^2^)] = 0.019
*wR*(*F* ^2^) = 0.035
*S* = 0.96
2095 reflections
245 parameters
2 restraints
H atoms treated by a mixture of independent and constrained refinement
Δρ_max_ = 0.17 e Å^−3^
Δρ_min_ = −0.22 e Å^−3^
Absolute structure: Flack (1983 ▶), 590 Friedel pairs
Flack parameter: 0.020 (7)

### 

Data collection: *CrysAlis PRO* (Oxford Diffraction, 2009 ▶); cell refinement: *CrysAlis PRO*; data reduction: *CrysAlis PRO*; program(s) used to solve structure: *SHELXS97* (Sheldrick, 2008 ▶); program(s) used to refine structure: *SHELXL97* (Sheldrick, 2008 ▶) within *WinGX* (Farrugia, 1999 ▶); molecular graphics: *PLATON* (Spek, 2009 ▶); software used to prepare material for publication: *PLATON*.

## Supplementary Material

Crystal structure: contains datablocks global, I. DOI: 10.1107/S1600536810025043/tk2684sup1.cif

Structure factors: contains datablocks I. DOI: 10.1107/S1600536810025043/tk2684Isup2.hkl

Additional supplementary materials:  crystallographic information; 3D view; checkCIF report

## Figures and Tables

**Table 1:** Hydrogen-bond geometry (Å, °)

*D*—H⋯*A*	*D*—H	H⋯*A*	*D*⋯*A*	*D*—H⋯*A*
O2—H21⋯N3*B*	0.99 (6)	1.77 (6)	2.753 (5)	180 (6)
N1*A*—H11*A*⋯O3	0.85 (3)	2.06 (3)	2.903 (3)	173 (3)
N1*A*—H12*A*⋯O1^i^	0.90 (3)	2.05 (3)	2.943 (4)	172 (3)
N2*A*—H21*A*⋯O2^i^	0.80 (3)	2.08 (3)	2.867 (4)	167 (3)
N2*A*—H22*A*⋯O3^ii^	0.94 (3)	2.00 (3)	2.925 (4)	167 (3)
N3*A*—H31*A*⋯O2^ii^	0.82 (3)	2.32 (3)	3.132 (3)	179 (5)
N3*A*—H32*A*⋯O1	0.95 (2)	1.91 (2)	2.859 (4)	174 (2)
N1*B*—H11*B*⋯O2*W*^iii^	0.89 (4)	2.34 (3)	3.151 (5)	151 (3)
N2*B*—H21*B*⋯O2*W*^iii^	0.88 (5)	2.18 (5)	3.026 (5)	163 (4)
N2*B*—H22*B*⋯O3	0.93 (4)	2.10 (4)	3.002 (4)	165 (3)
N3*B*—H31*B*⋯O2*W*^iv^	0.80 (3)	2.15 (3)	2.935 (5)	169 (4)
O1*W*—H11*W*⋯O3	0.95 (3)	1.81 (3)	2.737 (3)	167 (4)
O1*W*—H12*W*⋯O2^v^	0.88 (3)	1.85 (4)	2.715 (4)	168 (4)
O2*W*—H21*W*⋯O1^i^	0.78 (4)	1.93 (4)	2.701 (4)	171 (4)
O2*W*—H22*W*⋯O1*W*	0.78 (4)	2.01 (4)	2.673 (4)	143 (4)

Symmetry codes: (i) 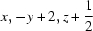; (ii) 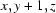; (iii) 
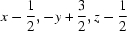
; (iv) 
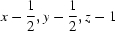
; (v) 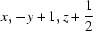.

**Fig. 1.**
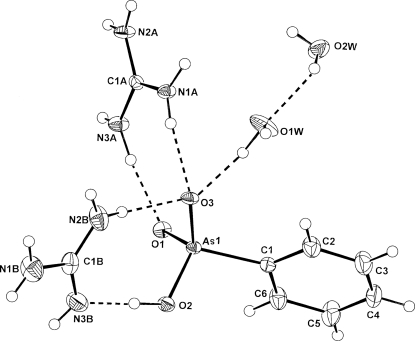
Molecular configuration and atom naming scheme for the guanidinium cation (A and the guanidine molecule B), the phenylarsonate anion and the two water molecules of solvation in (I). Inter-species hydrogen bonds are shown as dashed lines. Displacement ellipsoids are drawn at the 40% probability level.

**Fig. 2.**
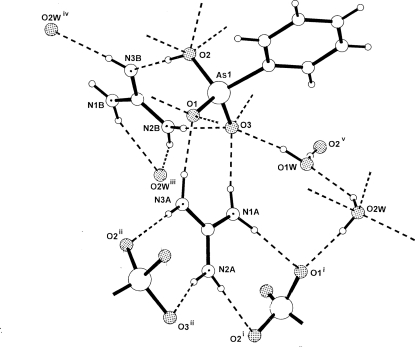
The hydrogen-bonding extensions of the basic asymmetric unit in the structure of (I), showing hydrogen-bonding associations as dashed lines. For symmetry codes, see Table 1.

**Fig. 3.**
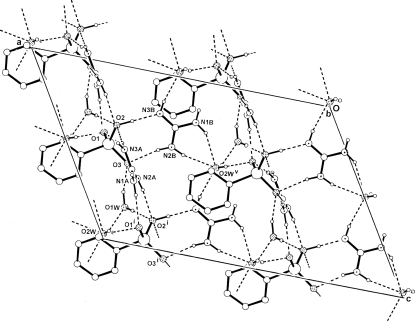
The hydrogen-bonded framework structure of (I) viewed down the b axial direction of the unit cell, showing hydrogen-bonding associations as dashed lines. Non-associative hydrogen atoms are deleted.

## supplementary crystallographic information

### Comment

The guanidinium cation has the capacity to form extended hydrogen-bonded structures through its six trigonally disposed H-donor sites. This ability is best illustrated in the host–guest clathrate structures with 4,4'-biphenyldisulfonate (Swift *et al.*, 1998, Swift & Ward, 1998) or the supramolecular rosette ribbons with HCO_3_^-^ and terephthalic acid (Mak & Xue, 2000). The hydrogen-bonded structures found in the guanidinium salts of carboxylic acids are largely three-dimensional and usually feature cyclic associations involving either two *N*–*H*···O_carboxyl_ links [graph set *R*_2_^2^(8) (Etter *et al.*, 1990)] or three-centre *N*–*H*···*O*,*O*'_carboxyl_ links [graph set *R*^2^_1_(6)]. Some examples of the structures of the guanidinium salts of monocyclic aromatic acids are those with pyromellitic acid (Sun *et al.*, 2002), 3,5-dinitrosalicylic acid (Smith *et al.*, 2001) and phenylacetic acid (Smith & Wermuth, 2010). This last compound has both types of cation-anion interaction but shows an unusual one-dimensional columnar structure. The structure of the guanidinium salt of phenylarsonic acid [benzenearsonic acid (O'Neil, 2001)] has not been previously reported.

Our 2:1 stoichiometric reaction of phenylarsonic acid with guanidinium carbonate aqueous propan-2-ol gave large, high-quality crystals of the title compound, the adduct hydrate CH_6_N_3_^+^ C_6_H_6_AsO_3_^-^. CH_5_N_3_. 2H_2_O (I), the structure of which is reported here.

In (I) the phenylarsonate anion gives two *R*_2_^2^(8) cyclic hydrogen-bonding interactions, one with a guanidinium cation (*A*), the other with a guanidine molecule (*B*), in which the second donor H atom is provided by the arsonate O–H group (Fig. 1). The anions are also bridged by the linked water molecules, one of which (O2W) completes a cyclic *R*_5_^3^(9) hydrogen-bonding association with a guanidinum cation (Fig. 2) (Table 1). This ring is conjoint with one of the three *R*_2_^2^(8) associations about the cation, whereas with the guanidine molecule there is one *R*_2_^2^(8) and one *R*^1^_2_(6) association, also with O2W. The overall result is a three-dimensional framework structure (Fig. 3).

It is notable that the As environment has a total of eight As–H contacts both inter- and intra-molecular with a range of 2.95 (3)–3.15 (3) Å. Also, one of the H atoms of the guanidine cation (H12B) has no feasibly situated acceptor atom.

### Experimental

The title compound was synthesized by heating together under reflux for 10 minutes, 1 mmol of phenylarsonic acid (benzenearsonic acid) and 0.5 mmol of guanidine carbonate in 50% aqueous propan-2-ol. After concentration to *ca* 30 ml, room temperature evaporation of the hot-filtered solution to moist dryness gave colourless plates of (I) (m.p. 505 K), from which a specimen suitable for X-ray analysis was cleaved.

### Refinement

Hydrogen atoms involved in hydrogen-bonding interactions were located by difference methods and their positional and isotropic displacement parameters were refined. The aromatic H atoms were included in the refinement in calculated positions (C–H = 0.93 Å) and treated as riding, with *U*_iso_(H) = 1.2*U*_eq_(C).

### Crystal data

**Table:**

CH_6_N_3_^+^·C_6_H_6_AsO_3_^−^·CH_5_N_3_·2H_2_O	*F*(000) = 736
*M**_r_* = 356.23	*D*_x_ = 1.548 Mg m^−^^3^
Monoclinic, *C**c*	Melting point: 505 K
Hall symbol: C -2yc	Mo *K*α radiation, λ = 0.71073 Å
*a* = 18.6545 (14) Å	Cell parameters from 3772 reflections
*b* = 7.6394 (3) Å	θ = 3.1–28.7°
*c* = 12.6319 (10) Å	µ = 2.25 mm^−^^1^
β = 121.856 (10)°	*T* = 200 K
*V* = 1529.0 (2) Å^3^	Block, colourless
*Z* = 4	0.27 × 0.25 × 0.20 mm

### Data collection

**Table:**

Oxford Diffraction Gemini-S CCD-detector diffractometer	2095 independent reflections
Radiation source: Enhance (Mo) X-ray source	1940 reflections with *I* > 2σ(*I*)
graphite	*R*_int_ = 0.024
Detector resolution: 16.08 pixels mm^-1^	θ_max_ = 26.0°, θ_min_ = 3.1°
ω scans	*h* = −22→21
Absorption correction: multi-scan (*SADABS*; Sheldrick, 1996)	*k* = −9→9
*T*_min_ = 0.935, *T*_max_ = 0.985	*l* = −11→15
4919 measured reflections	

### Refinement

**Table:**

Refinement on *F*^2^	Secondary atom site location: difference Fourier map
Least-squares matrix: full	Hydrogen site location: inferred from neighbouring sites
*R*[*F*^2^ > 2σ(*F*^2^)] = 0.019	H atoms treated by a mixture of independent and constrained refinement
*wR*(*F*^2^) = 0.035	*w* = 1/[σ^2^(*F*_o_^2^) + (0.0159*P*)^2^] where *P* = (*F*_o_^2^ + 2*F*_c_^2^)/3
*S* = 0.96	(Δ/σ)_max_ = 0.002
2095 reflections	Δρ_max_ = 0.17 e Å^−^^3^
245 parameters	Δρ_min_ = −0.22 e Å^−^^3^
2 restraints	Absolute structure: Flack (1983), 590 Friedel pairs
Primary atom site location: structure-invariant direct methods	Flack parameter: 0.020 (7)

### Special details

**Table:**

Geometry. Bond distances, angles *etc*. have been calculated using the rounded fractional coordinates. All su's are estimated from the variances of the (full) variance-covariance matrix. The cell e.s.d.'s are taken into account in the estimation of distances, angles and torsion angles
Refinement. Refinement of *F*^2^ against ALL reflections. The weighted *R*-factor *wR* and goodness of fit *S* are based on *F*^2^, conventional *R*-factors *R* are based on *F*, with *F* set to zero for negative *F*^2^. The threshold expression of *F*^2^ > σ(*F*^2^) is used only for calculating *R*-factors(gt) *etc*. and is not relevant to the choice of reflections for refinement. *R*-factors based on *F*^2^ are statistically about twice as large as those based on *F*, and *R*- factors based on ALL data will be even larger.

### Fractional atomic coordinates and isotropic or equivalent isotropic displacement parameters (Å^2^)

**Table:**

	*x*	*y*	*z*	*U*_iso_*/*U*_eq_	
As1	0.85472 (1)	0.69726 (3)	0.45636 (2)	0.0162 (1)	
O1	0.85598 (11)	0.8928 (2)	0.39683 (17)	0.0220 (6)	
O2	0.79876 (11)	0.5495 (2)	0.34181 (17)	0.0223 (6)	
O3	0.81253 (10)	0.7081 (2)	0.54592 (15)	0.0209 (6)	
C1	0.96902 (16)	0.6103 (3)	0.5496 (2)	0.0214 (8)	
C2	1.00375 (19)	0.5330 (4)	0.6658 (3)	0.0312 (10)	
C3	1.0834 (2)	0.4638 (5)	0.7248 (4)	0.0393 (12)	
C4	1.1311 (2)	0.4729 (5)	0.6705 (3)	0.0388 (11)	
C5	1.0978 (2)	0.5496 (5)	0.5556 (4)	0.0423 (16)	
C6	1.0164 (2)	0.6166 (5)	0.4950 (3)	0.0326 (11)	
N1A	0.81112 (15)	1.0554 (3)	0.6380 (3)	0.0214 (8)	
N2A	0.79667 (18)	1.3549 (4)	0.6207 (3)	0.0253 (9)	
N3A	0.80233 (15)	1.1956 (4)	0.4697 (2)	0.0259 (8)	
C1A	0.80313 (16)	1.2018 (4)	0.5759 (2)	0.0185 (8)	
N1B	0.50840 (19)	0.4864 (5)	0.2386 (4)	0.0452 (11)	
N2B	0.6296 (2)	0.6056 (4)	0.4032 (3)	0.0391 (12)	
N3B	0.6265 (2)	0.5171 (5)	0.2284 (4)	0.0391 (11)	
C1B	0.5899 (2)	0.5383 (4)	0.2899 (4)	0.0301 (11)	
O1W	0.88312 (19)	0.7021 (3)	0.7987 (2)	0.0416 (10)	
O2W	0.99736 (15)	0.9183 (4)	0.9723 (2)	0.0421 (8)	
H2	0.97270	0.52800	0.70410	0.0370*	
H3	1.10560	0.41020	0.80200	0.0470*	
H4	1.18560	0.42720	0.71170	0.0470*	
H5	1.12960	0.55670	0.51860	0.0510*	
H6	0.99350	0.66650	0.41640	0.0390*	
H21	0.737 (3)	0.538 (7)	0.301 (4)	0.042 (11)*	
H11A	0.8076 (16)	0.956 (4)	0.606 (3)	0.036 (8)*	
H12A	0.823 (2)	1.061 (4)	0.717 (3)	0.043 (10)*	
H21A	0.7921 (16)	1.368 (4)	0.680 (3)	0.038 (7)*	
H22A	0.7998 (16)	1.461 (4)	0.585 (3)	0.045 (8)*	
H31A	0.8017 (17)	1.287 (4)	0.436 (3)	0.035 (8)*	
H32A	0.8159 (13)	1.093 (3)	0.441 (2)	0.037 (6)*	
H11B	0.486 (2)	0.501 (5)	0.285 (3)	0.048 (11)*	
H12B	0.480 (2)	0.444 (5)	0.166 (4)	0.052 (12)*	
H21B	0.599 (2)	0.613 (5)	0.437 (4)	0.053 (12)*	
H22B	0.683 (2)	0.653 (4)	0.436 (3)	0.055 (9)*	
H31B	0.5958 (18)	0.495 (4)	0.156 (3)	0.050 (9)*	
H11W	0.8672 (19)	0.702 (4)	0.714 (3)	0.052 (9)*	
H12W	0.859 (2)	0.610 (4)	0.809 (3)	0.046 (10)*	
H21W	0.960 (2)	0.982 (5)	0.954 (3)	0.046 (12)*	
H22W	0.973 (2)	0.830 (5)	0.950 (4)	0.048 (12)*	

### Atomic displacement parameters (Å^2^)

**Table:**

	*U*^11^	*U*^22^	*U*^33^	*U*^12^	*U*^13^	*U*^23^
As1	0.0201 (1)	0.0152 (1)	0.0153 (1)	−0.0004 (2)	0.0107 (1)	−0.0003 (2)
O1	0.0286 (10)	0.0189 (10)	0.0231 (11)	−0.0003 (8)	0.0167 (9)	0.0020 (8)
O2	0.0206 (10)	0.0247 (10)	0.0215 (11)	−0.0028 (8)	0.0111 (8)	−0.0049 (8)
O3	0.0278 (10)	0.0226 (9)	0.0174 (10)	0.0002 (8)	0.0155 (8)	−0.0031 (8)
C1	0.0212 (14)	0.0191 (13)	0.0205 (15)	−0.0008 (11)	0.0087 (12)	−0.0026 (12)
C2	0.0302 (17)	0.0396 (18)	0.0248 (17)	0.0038 (14)	0.0153 (14)	0.0063 (14)
C3	0.034 (2)	0.051 (2)	0.027 (2)	0.0097 (18)	0.0121 (17)	0.0101 (18)
C4	0.0249 (19)	0.051 (2)	0.033 (2)	0.0132 (17)	0.0101 (15)	0.0051 (18)
C5	0.026 (2)	0.070 (3)	0.039 (3)	0.0116 (19)	0.0226 (19)	0.013 (2)
C6	0.0287 (19)	0.0407 (19)	0.0261 (18)	0.0037 (15)	0.0130 (14)	0.0105 (16)
N1A	0.0329 (14)	0.0147 (12)	0.0211 (14)	0.0015 (10)	0.0174 (12)	0.0009 (11)
N2A	0.0425 (18)	0.0191 (15)	0.0245 (16)	0.0001 (12)	0.0246 (14)	−0.0022 (12)
N3A	0.0440 (15)	0.0198 (12)	0.0223 (13)	0.0015 (12)	0.0233 (11)	0.0028 (12)
C1A	0.0165 (13)	0.0203 (14)	0.0167 (14)	−0.0022 (12)	0.0075 (11)	−0.0028 (13)
N1B	0.0262 (17)	0.061 (2)	0.046 (2)	−0.0091 (14)	0.0175 (16)	−0.0030 (17)
N2B	0.027 (2)	0.054 (2)	0.032 (2)	−0.0023 (15)	0.0127 (16)	0.0048 (15)
N3B	0.0340 (19)	0.049 (2)	0.031 (2)	−0.0045 (16)	0.0150 (17)	−0.0104 (17)
C1B	0.0252 (19)	0.027 (2)	0.034 (2)	−0.0006 (15)	0.0128 (19)	0.0063 (18)
O1W	0.068 (2)	0.0355 (17)	0.0222 (14)	−0.0275 (14)	0.0245 (14)	−0.0064 (13)
O2W	0.0281 (13)	0.0333 (14)	0.0532 (16)	0.0002 (12)	0.0134 (12)	−0.0182 (13)

### Geometric parameters (Å, °)

**Table:**

As1—O1	1.6781 (17)	N2B—C1B	1.320 (5)
As1—O2	1.6921 (17)	N3B—C1B	1.287 (6)
As1—O3	1.687 (2)	N1B—H11B	0.89 (4)
As1—C1	1.930 (3)	N1B—H12B	0.85 (4)
O2—H21	0.99 (6)	N2B—H21B	0.88 (5)
O1W—H11W	0.95 (3)	N2B—H22B	0.93 (4)
O1W—H12W	0.88 (3)	N3B—H31B	0.80 (3)
O2W—H21W	0.78 (4)	C1—C2	1.385 (4)
O2W—H22W	0.78 (4)	C1—C6	1.379 (5)
N1A—C1A	1.329 (4)	C2—C3	1.369 (6)
N2A—C1A	1.332 (4)	C3—C4	1.383 (6)
N3A—C1A	1.335 (3)	C4—C5	1.373 (5)
N1A—H12A	0.90 (3)	C5—C6	1.388 (6)
N1A—H11A	0.85 (3)	C2—H2	0.9300
N2A—H21A	0.80 (3)	C3—H3	0.9300
N2A—H22A	0.94 (3)	C4—H4	0.9300
N3A—H31A	0.82 (3)	C5—H5	0.9300
N3A—H32A	0.95 (2)	C6—H6	0.9300
N1B—C1B	1.360 (6)		
			
As1···H11A	3.16 (3)	As1···H32A	3.09 (2)
As1···H11W	3.14 (3)	As1···H12W^ii^	3.02 (3)
As1···H22A^i^	2.95 (3)	As1···H21A^iii^	3.08 (3)
As1···H22B	3.09 (4)	As1···H21W^iii^	3.15 (4)
			
O1—As1—O2	111.03 (9)	As1—C1—C6	118.4 (2)
O1—As1—O3	112.25 (9)	C2—C1—C6	118.7 (3)
O1—As1—C1	107.92 (11)	As1—C1—C2	122.8 (3)
O2—As1—O3	108.09 (10)	C1—C2—C3	120.5 (4)
O2—As1—C1	106.05 (10)	C2—C3—C4	120.6 (4)
O3—As1—C1	111.34 (10)	C3—C4—C5	119.7 (4)
As1—O2—H21	122 (3)	C4—C5—C6	119.6 (4)
H11W—O1W—H12W	107 (3)	C1—C6—C5	121.0 (3)
H21W—O2W—H22W	100 (4)	C3—C2—H2	120.00
H11A—N1A—H12A	119 (3)	C1—C2—H2	120.00
C1A—N1A—H11A	121 (2)	C2—C3—H3	120.00
C1A—N1A—H12A	120 (2)	C4—C3—H3	120.00
H21A—N2A—H22A	114 (3)	C5—C4—H4	120.00
C1A—N2A—H21A	126 (2)	C3—C4—H4	120.00
C1A—N2A—H22A	121 (2)	C6—C5—H5	120.00
H31A—N3A—H32A	116 (3)	C4—C5—H5	120.00
C1A—N3A—H32A	123.2 (14)	C5—C6—H6	119.00
C1A—N3A—H31A	119 (2)	C1—C6—H6	120.00
C1B—N1B—H11B	117 (2)	N2A—C1A—N3A	120.2 (3)
H11B—N1B—H12B	121 (4)	N1A—C1A—N2A	119.7 (3)
C1B—N1B—H12B	122 (3)	N1A—C1A—N3A	120.1 (3)
C1B—N2B—H22B	120 (2)	N2B—C1B—N3B	122.1 (4)
C1B—N2B—H21B	115 (3)	N1B—C1B—N2B	118.5 (4)
H21B—N2B—H22B	125 (4)	N1B—C1B—N3B	119.5 (4)
C1B—N3B—H31B	115 (3)		
			
O1—As1—C1—C2	136.3 (2)	C6—C1—C2—C3	−0.2 (5)
O1—As1—C1—C6	−47.5 (2)	As1—C1—C6—C5	−177.4 (3)
O2—As1—C1—C2	−104.7 (2)	C2—C1—C6—C5	−1.0 (5)
O2—As1—C1—C6	71.6 (2)	C1—C2—C3—C4	1.3 (5)
O3—As1—C1—C2	12.7 (2)	C2—C3—C4—C5	−1.0 (6)
O3—As1—C1—C6	−171.1 (2)	C3—C4—C5—C6	−0.3 (6)
As1—C1—C2—C3	176.0 (3)	C4—C5—C6—C1	1.3 (6)

Symmetry codes: (i) *x*, *y*−1, *z*; (ii) *x*, −*y*+1, *z*−1/2; (iii) *x*, −*y*+2, *z*−1/2.

### Hydrogen-bond geometry (Å, °)

**Table:**

*D*—H···*A*	*D*—H	H···*A*	*D*···*A*	*D*—H···*A*
O2—H21···N3B	0.99 (6)	1.77 (6)	2.753 (5)	180 (6)
N1A—H11A···O3	0.85 (3)	2.06 (3)	2.903 (3)	173 (3)
N1A—H12A···O1^iv^	0.90 (3)	2.05 (3)	2.943 (4)	172 (3)
N2A—H21A···O2^iv^	0.80 (3)	2.08 (3)	2.867 (4)	167 (3)
N2A—H22A···O3^v^	0.94 (3)	2.00 (3)	2.925 (4)	167 (3)
N3A—H31A···O2^v^	0.82 (3)	2.32 (3)	3.132 (3)	179 (5)
N3A—H32A···O1	0.95 (2)	1.91 (2)	2.859 (4)	174 (2)
N1B—H11B···O2W^vi^	0.89 (4)	2.34 (3)	3.151 (5)	151 (3)
N2B—H21B···O2W^vi^	0.88 (5)	2.18 (5)	3.026 (5)	163 (4)
N2B—H22B···O3	0.93 (4)	2.10 (4)	3.002 (4)	165 (3)
N3B—H31B···O2W^vii^	0.80 (3)	2.15 (3)	2.935 (5)	169 (4)
O1W—H11W···O3	0.95 (3)	1.81 (3)	2.737 (3)	167 (4)
O1W—H12W···O2^viii^	0.88 (3)	1.85 (4)	2.715 (4)	168 (4)
O2W—H21W···O1^iv^	0.78 (4)	1.93 (4)	2.701 (4)	171 (4)
O2W—H22W···O1W	0.78 (4)	2.01 (4)	2.673 (4)	143 (4)

Symmetry codes: (iv) *x*, −*y*+2, *z*+1/2; (v) *x*, *y*+1, *z*; (vi) *x*−1/2, −*y*+3/2, *z*−1/2; (vii) *x*−1/2, *y*−1/2, *z*−1; (viii) *x*, −*y*+1, *z*+1/2.
